# Comparative Performance of ChatGPT-5 and Gemini 2.5 on the Official Clinical Diabetology Specialty Examination

**DOI:** 10.7759/cureus.101731

**Published:** 2026-01-17

**Authors:** Anna Kowalczyk, Michalina Loson-Kawalec, Dawid Bartosik, Aleksander Tabor, Bartosz Starzynski, Patrycja Dadynska, Piotr Sawina, Marta Zerek, Gracjan Sitarek, Dawid Szymanski, Maciej Majchrzak, Mateusz Podkanowicz, Alicja Szalach, Katarzyna Romanowicz, Tomasz Dolata, Adrianna Pielech

**Affiliations:** 1 Faculty of Medicine, University of Opole, Opole, POL; 2 Department of Internal Medicine, Karol Marcinkowski University Hospital, Zielona Góra, POL; 3 Department of Internal Medicine, Non-Public Health Care Institution (NZOZ) Hospital, Dzierżoniów, POL; 4 Faculty of Medicine, University of Zielona Góra, Zielona Góra, POL; 5 Department of Internal Medicine, Multispecialty Independent Public Health Care Institution Hospital, Nowa Sól, POL; 6 Department of Internal Medicine, Lower Silesian Oncology Center, Wrocław, POL

**Keywords:** artificial intelligence in medicine, chatgpt-5, diabetology, gemini, large language models (llms), specialty examination

## Abstract

Introduction

Artificial intelligence (AI) is becoming progressively popular in so many parts of our lives, and medicine is not an exception. Many people from the medical and non-medical environments ask themselves, "Will it be better than the doctors?". To become a doctor in Poland, you have to pass many exams, which also include a specialization exam. How can AI cope with the Official Clinical Diabetology Specialty Test?

Objective

The purpose of this article was to show how AI tools, such as ChatGPT-5 (OpenAI, San Francisco, CA, USA) and Gemini 2.5 (Google DeepMind, London, UK), are handling the Official Clinical Diabetology Specialty Test of Poland. The first outcome was to compare the accuracy of the answers with the official answer key. Secondly, the result was the confidence of models in their responses. Both of the models were statistically compared using McNemar's test.

Methodology

The study analyzed 117 questions randomly chosen from the Centre for Medical Examination (CEM) archive in Łódź, Poland. The questions were multiple-choiced with one correct answer. ChatGPT-5 and Gemini 2.5 were responding to these questions in the Polish language. The assessment was based on the official key advertised on the CEM. Statistical analysis was executed using McNemar's test.

Results

ChatGPT-5 answered correctly for 92 questions (78.63%). On the other hand, Gemini 2.5 achieved an accuracy rate of 68.38%, because it gave correct answers in 80 of 117 questions. Collations of these two models received statistically relevant differences (χ² = 4.84; p = 0.0455). In an analysis of confidence in giving the answers, both models were on a similar level (GPT-5 = 5, while Gemini 2.5 = 4.957 on a five-point scale).

Conclusions

Scores, which reached both of the AI models, ChatGPT-5 and Gemini 2.5, allowed for passing the Official Clinical Diabetology Specialty Examination. The results are hopeful, because we can use AI in the study process, but it still has to be more adapted to manage clinical cases. We can use it as a tool in learning, but for now, it cannot step in for specialists.

## Introduction

Large language models (LLMs) have become key tools now widely used across many different fields. Medicine is no exception; these models are making their way into healthcare applications. Among the better-known examples are ChatGPT (OpenAI, San Francisco, CA, USA) and Gemini (Google DeepMind, London, UK), which generate responses that closely resemble natural human conversation. Their main function is to interpret and respond to fairly complex instructions [1]. What sets LLMs apart is that they're trained on vast amounts of data beforehand. This preparation helps them grasp complicated language patterns and adjust to a variety of tasks, which isn't something every program can pull off easily. Techniques like fine-tuning or prompting are often used to improve their performance [2].

ChatGPT, for instance, is a language model powered by deep learning. It came out first in 2022 mainly as a prototype aimed at gathering feedback from early users to figure out both its strengths and weaknesses [3,4]. OpenAI built in systems to identify and filter out inappropriate or unwanted content, thus shaping replies to be more suitable for different audiences [5]. The most recent iteration, GPT-5, was released on August 7, 2025 [6].

Gemini is an artificial intelligence (AI) tool introduced on December 6, 2023. It utilizes a system built on vision language model (VLM) technology and serves as a competitor to ChatGPT [7].

Gemini AI integrates data sources such as Google Search [8] to deliver relatively quick and deep analysis of the desired issue. This complex system is able to exploit context and transfer it into conversation [9]. In comparison to ChatGPT, Gemini can utilize many types of inputs, such as audio, images, or videos, to generate the desired answer [7].

In recent years, journals focused on diabetes findings introduced studies about AI applications in diabetes care, but were more adjusted around ChatGPT than Gemini [10]. A new study published in Clinical Diabetology on July 7, 2025, by researchers affiliated with Wroclaw Medical University, introduces the purpose of older models of ChatGPT in the Specialty Certificate Exam in Diabetology. The results suggested that neither of the AI models, ChatGPT-3.5 and ChatGPT-4o, achieved a passing score of 60% in all exams. Nevertheless, both systems were close to the passing threshold; the total score from all exams oscillated between 54.8% for ChatGPT-4o and 60.7% for ChatGPT-3.5 [11].

The aim of this study was to evaluate whether recently released LLMs, such as ChatGPT-5 or Gemini, were able to succeed and which of them performed better on the Official Clinical Diabetology Specialty Test. The following research shows the overall response accuracy of Gemini 2.5 and ChatGPT-5 according to the official key, as well as the mean confidence levels of these language models.

## Materials and methods

The study using the GPT-5 model and Gemini 2.5 model was conducted between October 14 and 16, 2025. The study focused on analyzing questions from the Spring 2025 Official Diabetology Specialty Test that were randomly selected from the Centre for Medical Examination (CEM) archive in Łódź, Poland. The examination questions and their official answers are openly available and readily accessible. An overall number of 120 multiple-choice questions were selected (Appendices). Each question included five possible answer options, of which only one was correct. The Examination Committee withdrew three questions from the originally selected 120. These questions were eliminated from the statistical analysis due to factual mistakes and inconsistencies with current medical knowledge. 

Prior to the exam, ChatGPT and Gemini were informed of the exam format, including the total number of questions and answer choices. In addition, after each question was entered into the chat, both models were asked to assign the level of confidence in the answer. A total of five options were available for selection: 1 (complete uncertainty), 2 (low certainty), 3 (moderate certainty), 4 (high certainty), and 5 (absolute certainty). Both chat sessions allowed only one attempt per question; the sessions remained continuous without resets between questions, and randomness settings were not controlled. The ChatGPT-5 and Gemini 2.5 applications answered questions in Polish. The analysis included 117 questions. Each question was asked separately, and after each response, the model was asked to rate the confidence of its answer. The accuracy of the answers was assessed using the official answer key published on the CEM website. Statistical analysis of the results was performed using McNemar's test. All the interactions with ChatGPT and Gemini, questions, and answers were documented. Every interaction with GPT-5 and Gemini 2.5 models took place entirely in the Polish language to ensure consistency with the Polish Official Diabetology Specialty Test. 

McNemar's test was conducted to demonstrate the difference in accuracy between the two models. P-values below 0.05 were considered statistically significant. 

Communication among the authors was carried out using online tools, including Google (Google LLC, Mountain View, CA, USA) and Facebook Messenger (Meta Platforms, Inc., Menlo Park, CA, USA).

Statistical analyses were conducted using Microsoft Excel (Microsoft Corp., Redmond, WA, USA) and GraphPad Prism 10 (GraphPad Software Inc., Boston, MA, USA).

Since the study did not involve human participants and private patient data, formal ethical approval was not required.

## Results

The ChatGPT-5 model provided 92 correct answers out of 117 questions. Three responses were found to be inconsistent with current knowledge and were therefore excluded. Consequently, ChatGPT-5 achieved an overall accuracy of 78.63%. In comparison, the Gemini 2.5 model delivered 80 correct answers, corresponding to an accuracy rate of 68.38%. The comparison between the two models, ChatGPT-5 and Gemini 2.5, revealed a statistically significant difference (χ² = 4.84; p = 0.0455) (Table 1). 

**Table 1:** Accuracy of GPT-5 and Gemini 2.5 on the Spring 2025 Diabetology PES Examination (n = 117) Data are presented as absolute numbers (n) and percentages (%). Subsequent comparative analysis using McNemar's test demonstrates a statistically significant difference in accuracy between the two models (χ² = 4.84; p = 0.0455). PES: National Specialty Examination

Model	Correct answers	Accuracy (%)	P-value	χ² value
GPT-5	92	78.63	0.0455	4.84
Gemini 2.5	80	68.38		

Analysis of the confidence ratings showed that both models performed consistently within a high confidence range. On a five-point scale, the average confidence for GPT-5 was 5, while Gemini 2.5 scored 4.957 (Table 2). 

**Table 2:** Comparison of mean confidence and standard deviations between GPT-5 and Gemini 2.5 Data are presented as absolute numbers (n).

Model	Mean confidence
GPT-5	5
Gemini 2.5	4.957

To further investigate the performance differences between the two models, a direct paired-response comparison was conducted. GPT-5 provided correct answers in 18 cases where Gemini 2.5 failed, while Gemini 2.5 outperformed GPT-5 in only seven cases. In 73 cases, both models provided correct answers, and in 17 cases, both provided incorrect answers (Table 3). 

**Table 3:** Contingency table comparing paired responses between GPT-5 and Gemini 2.5 on the Spring 2025 Diabetology PES Examination (n = 117) Data are presented as absolute numbers (n). Subsequent comparative analysis using McNemar's test demonstrates a statistically significant difference in accuracy between the two models (p = 0.0455; χ² = 4.84). PES: National Specialty Examination

	GPT-5 incorrect	GPT-5 correct	P-value	χ² value
Gemini 2.5 incorrect	17	18	0.0455	4.84
Gemini 2.5 correct	7	73		

## Discussion

ChatGPT-5 debuted on the market on August 7, 2025, as the successor to ChatGPT-4o. It introduced numerous improvements over previous versions, such as shorter response times, enhanced coding and writing abilities, and a reduced level of model hallucinations. OpenAI also emphasizes that this model provides much more critical and reflective answers. According to its creators, GPT-5 is the best model to date for answering questions related to various fields of healthcare [12]. 

In this study, the model presented above was compared to Gemini 2.5, developed by Google DeepMind, which was released on March 25, 2025. Gemini 2.5 is a "thinking" model capable of performing reasoning before responding to a given question. As a result, it is expected to deliver higher performance and greater accuracy in its answers [13]. 

ChatGPT shows potential in several areas, including clinical decision support, medical education through interactive simulations, data analysis for disease pattern recognition, and patient communication regarding prevention and treatment preparation.

Moreover, the integration of such tools into medical education enables faster knowledge acquisition and supports continuous learning among healthcare professionals. With proper oversight and adherence to ethical standards, ChatGPT can serve as a valuable complement to medical practice and specialist training, contributing to greater efficiency and accessibility of healthcare [14,15].

Both models were evaluated for their effectiveness in answering advanced medical questions sourced from the resources of the CEM in Łódź, used for the purposes of the National Specialty Examination (PES) in Diabetology, which consists of 120 questions [16]. Due to factual errors and inconsistencies with current medical knowledge, three questions were removed and excluded from the statistical analysis.

During the test, ChatGPT-5 provided 92 correct answers, representing 78.63%, while Gemini 2.5 provided 80 correct answers, representing 68.38% of all responses.

The study therefore showed that ChatGPT-5 performed better on the specialization exam than Gemini 2.5; a detailed data analysis revealed a statistically significant difference between the results. Nevertheless, both models managed to achieve a passing score on the exam (passing threshold: 60%).

In earlier studies comparing the ChatGPT-3.5 and ChatGPT-4o models [9] in solving the PES in Diabetology, the chatbots completed three exams (Autumn 2023, Spring 2023, and Autumn 2024). ChatGPT-3.5 achieved 218 correct answers out of 359 questions (60.7%), while ChatGPT-4o achieved 197 correct answers (54.8%). No significant differences were found between these models; however, in the present study, ChatGPT-5 demonstrates an increase in effectiveness compared to its predecessors (78.63%), indicating that its potential in supporting medical education is also growing.

The Gemini model has not previously been tested in solving the PES in Diabetology; therefore, this study represents the first attempt to assess its effectiveness. However, a comparative analysis of the performance of ChatGPT-4o and Gemini Advanced in training exams for diagnostic radiology [17] showed the superiority of the OpenAI model (69.8%) over Google's solution (60.4%). The results of our study on the newer versions of these models indicate that the OpenAI model continues to maintain an advantage in effectively answering advanced medical questions.

The results obtained in our study can be compared to a previously conducted study that compared ChatGPT-5's answers with the official PES results in child and adolescent psychiatry [18]. In that study, GPT-5 correctly answered 97 out of 120 questions, representing 80.8% of all responses, thus achieving a passing score and significantly exceeding the 60% threshold. These findings are also consistent with the study by Latkowska et al., who evaluated ChatGPT-5's ability to pass the specialization exam in endocrinology [19]. In their case, ChatGPT-5 achieved a score of 76.47%. Overall, the GPT-5 model demonstrates passing performance in the range of approximately 75-80%.

It is worth noting that our study was conducted based on a single specialization exam and the pool of such exams available in Polish remains limited, which makes it difficult to compare our results with other publications. Irrespective of this fact, the work presented herein is one of the first of its kind to be conducted in Poland and one of the very few internationally that has systematically compared the performance of the newest LLM in the field of advanced diabetology knowledge. It is also the first study to directly compare the new GPT-5 model with Gemini 2.5 in this domain.

Our findings constitute a valuable contribution to the growing field of research on the application of AI in medical education and practice, especially in the context of the Polish language, which up to now has been underrepresented in studies analyzing the performance of LLMs. The study not only confirms the potential of GPT-5 to support specialist medical training but also serves as a reference point for future comparative analyses involving other AI models.

Further research is necessary because, to draw high-quality conclusions, a large number of tests need to be analyzed, thereby reducing the influence of extreme results on statistical analyses. It should also be kept in mind that the models studied receive training data primarily in English, which may significantly limit their performance when working in Polish.

Additionally, it should be noted that the language models examined are not trained exclusively to solve advanced medical tests, which may substantially constrain their potential. It is expected that in the future, domain-specific models will be developed, trained solely in narrowly specialized fields and in various languages, thereby increasing their potential to support medical education.

The past few years have seen dynamic development in language models, which continue to surpass successive barriers, such as passing the Turing test [20]. Particularly promising for the advancement of medicine are the contributions of Nobel Prize winners in Chemistry, Demis Hassabis and John Jumper [21], who, with their AI-based AlphaFold2 model, achieved a breakthrough in designing and predicting the three-dimensional structure of proteins.

It is worth bearing in mind that the answers generated by AI may not always be completely complete or accurate, so it is recommended to verify them before making diagnostic decisions. In the case of an inaccurate diagnosis, the issue of responsibility also arises; it must be determined whether the error lies with the program itself, its creators, or the individual using it [22,23]. 

Despite these limitations, the development of language models such as ChatGPT or Gemini represents one of the most promising advances in modern digital medicine. These systems are increasingly applied as intelligent clinical assistants, supporting physicians in data analysis, the interpretation of test results, literature review, and the preparation of diagnostic and therapeutic plans.

## Conclusions

In the study in question, the ChatGPT-5 model answered 92 questions out of 120 correctly, which translates to a score of 78.63%. On the other hand, Gemini 2.5 gave 80 correct answers out of 120, corresponding to 68.38%. The passing threshold to qualify for the oral part of PES is 60%, so both models passed that threshold. Thus, Gemini 2.5 qualified for the oral stage. With a score of 78.63%, ChatGPT-5 exceeded the threshold of 75%, which, under the rules of the examination, means exemption from the oral part.

The study shows that the language models assessed in this work offer meaningful possibilities for use in medical education, though their outputs still require careful interpretation and cannot replace expert judgment. Ongoing, expert-led evaluation remains crucial for minimizing errors and ensuring safety in educational settings. At the same time, the results suggest that, when used responsibly and under appropriate oversight, AI can serve as a helpful aid in physicians' professional development and in improving the accessibility and quality of specialized medical training.

## Appendices

**Table 4:** Comparison of GPT-5 and Gemini 2.5 responses to questions from the Spring 2025 Diabetology Specialty Examination (PES) with declared confidence levels The table presents the responses of GPT-5 and Gemini 2.5 compared to the correct answers (A-E), based on the official answer key published by the Medical Examination Center (CEM) in Łódź. For each question, both GPT-5 and Gemini 2.5 declared their self-assessed confidence level which is reported on a scale ranging from 1 to 5 (1 = complete uncertainty; 5 = absolute certainty). PES: National Specialty Examination

Question number	Question	Answers	Answer according to CEM key	Gemini 2.5 answer	GPT-5 answer	Confidence level 1-5 of Gemini 2.5	Confidence level 1-5 of GPT-5
1	In an unconscious patient with a blood glucose level of 59 mg/dl (glucometer measurement), one should diagnose:	A. Mild hypoglycemia and administer glucose orally.	C	C	C	5	5
		B. Mild hypoglycemia and administer 40% glucose intravenously.					
		C. Severe hypoglycemia and administer 20% glucose intravenously.					
		D. Severe hypoglycemia and administer 4 ampoules of 40% glucose orally.					
		E. The patient does not meet the criteria for severe hypoglycemia.					
2	A 63-year-old patient with type 2 diabetes for 13 years, with HbA1c 7.6%, obesity, no cardiovascular complications, GFR 56 ml/min, treated with: glimepiride 4 mg/day, metformin 3g/day, semaglutide s.c. 1 mg weekly, requires: (1) lifestyle advice; (2) increasing the dose of glimepiride to 2×4 mg; (3) changing metformin to an extended-release form; (4) adding an SGLT-2 inhibitor to the current therapy; (5) adding basal insulin at bedtime to the therapy. The correct answer is:	A. 1, 4.	A	A	B	5	5
		B. 1, 3, 4.					
		C. 2, 3, 4.					
		D. 1, 2.					
		E. 1, 5.					
3	Glucagon can be administered to treat hypoglycemia because: (1) it stimulates hepatic gluconeogenesis; (2) it inhibits hepatic gluconeogenesis; (3) it stimulates glycogenolysis; (4) it inhibits glycogenolysis; (5) administered in a dose of 1 mg i.m., it increases blood glucose levels by 50 mg/dl. The correct answer is:	A. 1, 3, 5.	A	A	A	5	5
		B. 2, 3, 5.					
		C. 1, 2.					
		D. Only 1.					
		E. 1, 4.					
4	Lactic acidosis should be suspected if there is: (1) rapidly progressing consciousness disorders with accompanying acidotic breathing and deep metabolic acidosis; (2) anion gap >10 mmol/l; (3) anion gap >20 mmol/l; (4) blood lactate concentration >3 mmol/l; (5) blood lactate concentration >7 mmol/l. The correct answer is:	A. 1, 2, 4.	C	C	C	5	5
		B. 1, 2, 5.					
		C. 1, 3, 5.					
		D. 2, 4.					
		E. 3, 4.					
5	Indicate the product that contains 10 g of carbohydrates (=1 carbohydrate exchange):	A. 100 g of sunflower seeds.	D	D	D	5	5
		B. 2 eggs.					
		C. 50 ml of orange juice.					
		D. 100 ml of a drink containing 10 g of carbohydrates.					
		E. A wheat roll weighing 60 g.					
6	Which of the following drugs requires discontinuation if GFR is below 30 ml/min/1.73 m^2^?:	A. Metformin.	A	A	A	5	5
		B. NPH insulin.					
		C. Dapagliflozin.					
		D. Empagliflozin.					
		E. Semaglutide.					
7	An OGTT with 75 g of glucose in a 35-year-old woman with gestational diabetes, hypertension, and obesity should be performed:	A. Between the 24th and 28th week of pregnancy.	E	A	E	5	5
		B. Between the 14th and 18th week of pregnancy.					
		C. Between the 13th and 10th week of pregnancy.					
		D. Between the 7th and 9th week of pregnancy.					
		E. At the time of pregnancy diagnosis.					
8	A drug that can be used until the end of the first trimester in a patient with diabetes is:	A. Metformin.	A	A	A	5	5
		B. Oral semaglutide.					
		C. Semaglutide in s.c. injections.					
		D. Dapagliflozin.					
		E. Empagliflozin.					
9	An absolute indication for insulin therapy is: (1) type 1 diabetes; (2) gestational diabetes treated with diet, if fasting glucose remains above 90 mg/dl, and 1-hour postprandial glucose is up to 140 mg/dl; (3) gestational diabetes treated with diet, if fasting glucose is up to 90 mg/dl, and 1-hour postprandial glucose is up to 140 mg/dl; (4) pregnancy in a woman with type 2 diabetes treated with semaglutide before pregnancy; (5) glucose level above 300 mg/dl, polyuria, and polydipsia. The correct answer is:	A. 1, 4.	D	A	B	5	5
		B. 1, 5.					
		C. 2, 4.					
		D. 1, 2, 4, 5.					
		E. 1, 3, 4, 5.					
10	The target glucose value to be aimed for in a diabetic patient hospitalized for an acute condition, such as myocardial infarction or stroke, is:	A. 100-180 mg/dl.	A	A	A	5	5
		B. 100-200 mg/dl.					
		C. 100-250 mg/dl.					
		D. 70-140 mg/dl.					
		E. 80-110 mg/dl.					
11	Clinically significant hypoglycemia is:	A. Hypoglycemia associated with severe cognitive impairment.	E	E	E	5	5
		B. Blood glucose below 70 mg/dl.					
		C. Blood glucose below 65 mg/dl.					
		D. Blood glucose below 60 mg/dl.					
		E. Blood glucose below 54 mg/dl.					
12	Treatment of hypoglycemia in a conscious person requires the administration of 15 g of easily digestible carbohydrates and a glucose level check after:	A. 5 minutes.	C	C	C	5	5
		B. 10 minutes.					
		C. 15 minutes.					
		D. 25 minutes.					
		E. 45 minutes.					
13	Weight reduction in a diabetic patient should occur at a rate of:	A. 0.5 kg per week.	A	A	A	5	5
		B. 2 kg per week.					
		C. 3 kg per week.					
		D. 4 kg per week.					
		E. 5 kg per week.					
14	A 21-year-old patient presented with the following test results: glucose in an oral glucose tolerance test (75 g glucose) - fasting 101 mg/dl, at the 2nd hour of the test 260 mg/dl, glucosuria in urine test, blood C-peptide concentration 1.2 ng/ml. Body weight 60 kg, height 172 cm. In her history: mother and maternal grandmother have diabetes. The most likely diagnosis is diabetes:	A. Type 1.	C	C	C	5	5
		B. Type 2.					
		C. MODY due to a mutation in the HNF1A gene.					
		D. MODY due to a mutation in the glucokinase gene.					
		E. Mitochondrial.					
15	What pharmacological treatment can be proposed in the first instance for neonatal diabetes associated with a mutation in the ABCC8 gene?:	A. Flozin.	B	B	B	5	5
		B. Sulfonylurea derivative.					
		C. Insulin.					
		D. Metformin.					
		E. Glitazone.					
16	Intense physical exercise can have an adverse effect on a person with diabetes:	A. Complicated by proliferative retinopathy.	E	A	E	5	5
		B. Complicated by autonomic neuropathy.					
		C. Diabetic nephropathy.					
		D. Answers A and C are correct.					
		E. Answers A, B, and C are correct.					
17	A 68-year-old patient with type 2 diabetes for 4 years, treated with metformin 3×850 mg by a primary care physician, came to a diabetologist for a consultation. In self-monitoring of blood glucose: fasting around 150 mg/dl, 2 hours after meals up to 250 mg/dl. In additional tests: HbA1c 7.9%, glucose 169 mg/dl, creatinine 1.6 mg/dl, GFR 42 ml/min/1.73 m2, potassium 4.6 mmol/l, total cholesterol 205 mg/dl, HDL 42 mg/dl, LDL 128 mg/dl, TG 205 mg/l. Indicate the correct course of action:	A. Reduce the metformin dose to 2×500 mg, add dapagliflozin 10 mg/day.	A	B	B	5	5
		B. Reduce the metformin dose to 2×850 mg, add dapagliflozin 10 mg/day.					
		C. Do not reduce the metformin dose, add dapagliflozin 10 mg/day.					
		D. Do not reduce the metformin dose, add sitagliptin 100 mg/day.					
		E. Discontinue metformin, use sitagliptin 100 mg/day.					
18	According to the recommendations of the Polish Diabetes Association (PTD), alcohol consumption:	A. Is absolutely contraindicated in people with type 2 diabetes.	C	E	E	5	5
		B. May promote hypoglycemia only in people treated with insulin.					
		C. The permissible amount of alcohol for a woman with type 2 diabetes is no more than 20 g (in terms of pure ethanol)/day.					
		D. The permissible amount of alcohol for a man with type 2 diabetes is no more than 40 g (in terms of pure ethanol)/day.					
		E. Answers C and D are correct.					
19	Diabetes related to cystic fibrosis:	A. Is most often diagnosed in the neonatal period.	D	E	D	5	5
		B. Is associated with a high risk of normoglycemic ketoacidosis.					
		C. Should be treated with a sulfonylurea derivative.					
		D. Should be treated with insulin.					
		E. Answers B and D are correct.					
20	Treatment with tirzepatide is contraindicated:	A. With a GFR below 45 ml/min/1.73 m2.	B	B	B	5	5
		B. In the 1st trimester of pregnancy.					
		C. In heart failure class II according to NYHA.					
		D. Answers A and B are correct.					
		E. Answers A, B, and C are correct.					
21	In women of childbearing age treated with tirzepatide:	A. Contraception is not recommended.	B	B	D	5	5
		B. An intrauterine device with progestogen activity may be used.					
		C. The use of combined oral hormonal contraception (estrogen-progestogen) is contraindicated.					
		D. Answers B and C are correct.					
		E. Answers A, B, and C are correct.					
22	In women with diabetes in pregnancy:	A. The target systolic blood pressure values are 110-139 mmHg.	E	C	E	5	5
		B. The target diastolic blood pressure values are 81-85 mmHg.					
		C. Methyldopa may be used in the treatment of hypertension.					
		D. Labetalol may be used in the treatment of hypertension.					
		E. All of the above.					
23	In a person being treated for ketoacidosis with an intravenous insulin infusion, it is necessary to:	A. Not administer potassium if the serum potassium concentration is >5.2 mmol/l.	E	D	D	5	5
		B. Administer 5-10 mmol/h of KCl if the serum potassium concentration is 5-5.5 mmol/l.					
		C. Administer 10-15 mmol/h of KCl if the serum potassium concentration is 4-5 mmol/l.					
		D. Answers A and C are correct.					
		E. Answers B and C are correct.					
24	After bariatric surgery, it is recommended to assess the results of the surgical treatment of type 2 diabetes and accompanying diseases. The following values indicate their remission after discontinuation of pharmacotherapy:	A. Triglyceride concentration <2.6 mmol/L, total cholesterol concentration <4 mmol/L, LDL cholesterol concentration <2.2 mmol/L, and HbA1c value <6.0%.	C	E	E	5	5
		B. Triglyceride concentration <2.6 mmol/L, total cholesterol concentration <5 mmol/L, LDL cholesterol concentration <2.6 mmol/L, and HbA1c value <5.7%.					
		C. Triglyceride concentration <2.2 mmol/L, total cholesterol concentration <4 mmol/L, LDL cholesterol concentration <2 mmol/L, and HbA1c value <6.5%.					
		D. Triglyceride concentration <2.2 mmol/L, total cholesterol concentration <6.4 mmol/L, LDL cholesterol concentration <4 mmol/L, and HbA1c value <6.5%.					
		E. Triglyceride concentration <2.2 mmol/L, total cholesterol concentration <5 mmol/L, LDL cholesterol concentration <2.2 mmol/L, and HbA1c value <6.0%.					
25	An absolute contraindication for qualifying patients with type 2 diabetes for metabolic surgery is:	A. The period of 36 months preceding a planned pregnancy, breastfeeding.	D	D	D	5	5
		B. A history of myocardial infarction.					
		C. Gilbert's syndrome.					
		D. Lack of acceptance of surgical treatment for type 2 diabetes by the patient.					
		E. Severe hypertriglyceridemia.					
26	In drivers applying for or holding a driving license of categories AM, A1, A2, A, B1, B, B+E, or T, a relative health contraindication to driving is:	A. Intensive insulin therapy.	D	D	D	5	5
		B. HbA1c value above 8%.					
		C. Use of CGM.					
		D. At least two cases of severe hypoglycemia in the last 12 months.					
		E. Sufficient patient knowledge regarding self-monitoring of diabetes.					
27	Surgical treatment of obesity should be recommended for people with type 2 diabetes and BMI >35 kg/m2, especially with coexisting other diseases and unsatisfactory glycemic control when using behavioral therapy and anti-glycemic drugs, according to the following principle:	A. Qualification for metabolic surgery is also recommended for any patient with BMI >30 kg/m2, type 2 diabetes, and coexisting Cushing's syndrome.	B	B	B	5	5
		B. People with type 2 diabetes between the ages of 18 and 65 are eligible for metabolic surgery, with the upper age limit being extendable to 70 years in justified cases.					
		C. A patient after surgical treatment of type 2 diabetes should remain under the care of a diabetologist and general surgeon for 12 months.					
		D. An absolute contraindication for qualifying patients with type 2 diabetes for bariatric surgery is insulin therapy.					
		E. For patients after surgical treatment of obesity, becoming pregnant is absolutely contraindicated, regardless of the time after the procedure.					
28	Relative health contraindications for jobs requiring higher health standards, where a doctor authorized for employee health check-ups may predict improvement, for patients with diabetes include: (1) lack of glycemic self-monitoring or low ability to control it; (2) lack of metabolic control of the disease (HbA1c ≥8%); (3) lack of metabolic control of the disease (HbA1c ≥7%); (4) insufficient patient knowledge about diabetes, hypoglycemia, and ways to prevent it; (5) non-compliance with medical recommendations. The correct answer is:	A. 1, 2, 4, 5.	A	A	A	5	5
		B. 1, 3, 4, 5.					
		C. 1, 2, 4.					
		D. 1, 3, 4.					
		E. 3, 5.					
29	According to the recommendations of the Polish Society of Endocrinology (PTE) and the Polish Diabetes Association (PTD) regarding screening for thyroid dysfunction in patients with type 2 diabetes: (1) during each visit to a diabetologist, a clinical examination for thyroid diseases is necessary; (2) in every patient with newly diagnosed type 2 diabetes, it is recommended to measure the TSH concentration; (3) in a patient with a TSH concentration from 2.5 mIU/l to the upper limit of the reference value, the TPO Ab titer should be measured; (4) in a patient with a TPO Ab titer above the reference values and a TSH concentration from 2.5 mIU/l to the upper limit of the reference value, it is recommended to measure the FT4 concentration, and also to repeat the TSH concentration test once a year; (5) in a patient with an imbalanced lipid profile, it is recommended to measure the TSH concentration. The correct answer is:	A. All of the above.	A	B	A	5	5
		B. 2, 5.					
		C. 1, 2, 3.					
		D. 1, 3, 4.					
		E. 2, 3.					
30	When should a 75 g OGTT be performed in a pregnant woman with type 2 diabetes?:	A. After the 32nd week of pregnancy.	E	D	E	5	5
		B. Between the 24th and 28th week of pregnancy.					
		C. Between the 14th and 18th week of pregnancy.					
		D. During the first visit to the doctor.					
		E. It should not be performed.					
31	What can be a secondary cause of hyperlipidemia? (1) alcohol; (2) diabetes; (3) hypothyroidism; (4) anorexia nervosa; (5) oral estrogens; (6) psoriasis; (7) systemic lupus erythematosus. The correct answer is:	A. All of the above.	A	C	C	5	5
		B. 1, 2, 3, 6, 7.					
		C. 1, 2, 3, 5, 6, 7.					
		D. 1, 2, 3, 5.					
		E. 4, 5, 7.					
32	In a 58-year-old patient with type 2 diabetes treated orally with a flozin, hypoglycemia may occur if:	A. The patient performs unexpected physical exertion.	D	D	D	5	5
		B. The patient skips a main meal during the day.					
		C. It is used in combination with metformin.					
		D. It is used in combination with gliclazide.					
		E. The patient reduces their body weight by more than 10% from the initial body weight.					
33	Which situation can cause diabetic ketoacidosis? (1) pregnancy; (2) alcohol abuse by a patient with type 1 diabetes; (3) taking a flozin by a patient with type 1 diabetes; (4) air in the infusion set of a personal insulin pump; (5) disconnecting the insulin pump for a full day of mountain climbing. The correct answer is:	A. All of the above.	A	A	A	5	5
		B. 1, 2, 4.					
		C. 2, 4, 5.					
		D. 4, 5.					
		E. 1, 2, 5.					
34	In which situation is it not necessary to temporarily or permanently stop using metformin?:	A. Current glomerular filtration rate of 25 ml/min/1.73 m2.	E	E	E	5	5
		B. Decompensation of heart failure.					
		C. Exacerbation of chronic obstructive pulmonary disease with coexisting respiratory failure.					
		D. Planned surgery.					
		E. Lactation period.					
35	In a 64-year-old patient with a 15-year history of type 2 diabetes, with symptoms of gastroparesis, it is recommended to: (1) use prokinetic drugs (e.g., cisapride, trimebutine); (2) eat smaller, more frequent meals; (3) use a phosphodiesterase type 5 inhibitor; (4) use proton pump blockers; (5) add salt to meals. The correct answer is:	A. 1, 2, 3, 4.	C	C	C	5	5
		B. 1, 3.					
		C. 1, 2, 4.					
		D. 1, 2.					
		E. 2, 3, 4, 5.					
36	Which of the antihyperglycemic drugs do not cause weight gain? (1) pioglitazone; (2) sulfonylurea derivatives; (3) DPP-4 inhibitors; (4) SGLT-2 inhibitors; (5) GLP-1 receptor agonists. The correct answer is:	A. Only 4.	C	C	C	5	5
		B. 4, 5.					
		C. 3, 4, 5.					
		D. 1, 2.					
		E. 1, 4, 5.					
37	In a 34-year-old CIIPI patient (30th week of pregnancy) with diabetes and hypertension up to 155/95 mmHg, the oral drug of choice is: (1) methyldopa; (2) ramipril; (3) labetalol; (4) verapamil; (5) telmisartan. The correct answer is:	A. All of the above.	D	C	C	5	5
		B. 2, 4.					
		C. 1, 3.					
		D. 1, 3, 4.					
		E. 2, 4, 5.					
38	Recommendations for physical exercise for a 30-year-old CIPI patient in the 25th week of pregnancy with gestational diabetes do not include:	A. Undertaking moderate-intensity aerobic physical exercise for at least 150 minutes per week (3-4 times a week for 30-60 minutes).	D	D	D	5	5
		B. Walking, water aerobics, stretching exercises are permissible.					
		C. Physical activity contributes to reducing the percentage of gestational diabetes, premature births, and cesarean sections.					
		D. An indication for delivery by cesarean section is a patient with gestational diabetes who is physically active.					
		E. Physical exercise (without medical contraindications) is an essential element of managing gestational diabetes.					
39	In a patient with type 2 diabetes who has not reached the target LDL-C level with statin therapy, combined lipid-lowering therapy uses a combination of drugs, except:	A. Statin + ezetimibe.	E	E	E	5	5
		B. Statin + ezetimibe + inclisiran-siRNA.					
		C. Statin + ezetimibe + alirocumab.					
		D. Statin + eicosapentaenoic acid ethyl ester (EPA).					
		E. Statin + aliskiren.					
40	A contraindication to the use of a statin is not:	A. Breastfeeding period.	D	E	E	5	5
		B. Childbearing period, if the woman does not use effective contraception.					
		C. Active viral hepatitis.					
		D. Any initial creatine kinase value above the upper limit of normal.					
		E. Choroideremia.					
41	In a 32-year-old CIIPII patient in the 28th week of pregnancy with gestational diabetes, the target fasting and pre-meal glucose value in self-monitoring with a glucometer is:	A. Below 140 mg/dl.	E	E	E	5	5
		B. Below 120 mg/dl.					
		C. Below 110 mg/dl.					
		D. 90-100 mg/dl.					
		E. 70-90 mg/dl.					
42	In a 32-year-old CIIPII patient in the 28th week of pregnancy with gestational diabetes, the maximum glucose value in the 1st hour after starting a meal in self-monitoring with a glucometer is:	A. Below 180 mg/dl.	D	B	B	5	5
		B. Below 160 mg/dl.					
		C. Below 100 mg/dl.					
		D. 110-140 mg/dl.					
		E. 70-90 mg/dl.					
43	Contraindications for NHF reimbursement of a personal insulin pump for people with diabetes include: (1) non-compliance with personal hygiene rules; (2) two episodes of ketoacidosis within a year; (3) severe, rapidly progressing proliferative retinopathy; (4) alcohol addiction of a parent of an 18-year-old patient; (5) a 35-year-old patient with type 1 diabetes. The correct answer is:	A. 1, 2, 3, 5.	A	A	A	5	5
		B. 2, 3, 5.					
		C. 2, 4.					
		D. Only 5.					
		E. Only 2.					
44	Correct glucose targets for a patient with type 1 diabetes in the 3rd trimester of pregnancy are:	A. HbA1c level >6.5%, in CGM glucose levels >70% in the range of 70-180 mg/dl.	D	D	D	5	5
		B. HbA1c level >6.5%, in CGM glucose levels <70% in the range of 70-180 mg/dl.					
		C. HbA1c level <6.0%, in CGM glucose levels >70% in the range of 65-140 mg/dl.					
		D. HbA1c level <6.0%, in CGM glucose levels >70% in the range of 63-140 mg/dl.					
		E. None of the above.					
45	In an overweight patient, a chronic smoker, with atherosclerotic changes in the coronary arteries confirmed by imaging, a laboratory test showed a glycated hemoglobin level of 6.3%, and a creatinine level of 64 μmol/l and GFR of 47 ml/min/1.73 m2. A decision to initiate therapy with:	A. Metformin + simvastatin.	E	E	E	5	5
		B. Metformin + sitagliptin.					
		C. Empagliflozin + semaglutide.					
		D. Dapagliflozin.					
		E. Answers C and D are correct.					
46	In the case of elderly people with long-standing diabetes and significant macroangiopathy complications (history of myocardial infarction and/or stroke) and/or numerous comorbidities, the glycemic target is:	A. 85% of glucose levels in the range of 70-180 mg/dl with CGM systems.	C	C	B	5	5
		B. 75% of glucose levels in the range of 70-180 mg/dl with CGM systems.					
		C. 50% of glucose levels in the range of 70-180 mg/dl with CGM systems.					
		D. 25% of glucose levels in the range of 70-180 mg/dl with CGM systems.					
		E. In such a patient, there is no need to assess glucose levels with CGM.					
47	The strongest antidiabetic drug with a very high potency, lowering glucose levels is:	A. Metformin.	E	E	E	5	5
		B. Gliclazide.					
		C. Empagliflozin.					
		D. Glargine.					
		E. Tirzepatide.					
48	A diagnosis of diabetes should be made in a patient with the following results, except:	A. A random glucose level of 230 mg/dl, measured by the patient with a glucometer.	X	X	X	X	X
		B. A fasting glucose level of 120 mg/dl, measured by the patient with a glucometer.					
		C. A glycated hemoglobin level of ≥6.5%.					
		D. A glucose level of 180 mg/dl (10.0 mmol/l) at the 1st hour of an OGTT 75 g, measured by a reference method.					
		E. A glucose level of 240 mg% at the 2nd hour of an OGTT.					
49	Indicate the false statement regarding the therapy of an obese patient:	A. Obesity is an epidemic of the 21st century.	C	C	C	5	5
		B. Achieving and maintaining a weight reduction of 10% causes remission of diabetes.					
		C. The implementation of nutritional therapy should be based on a reduction in the caloric content of the diet, so that the total caloric content is 800-1000 kcal/day.					
		D. Metabolic surgery causes an average weight reduction of over 20%, leading to remission of diabetes, improvement of cardiovascular events, and reduction of all-cause mortality.					
		E. In patients with type 2 diabetes and overweight or obesity, weight reduction is a treatment priority.					
50	A 48-year-old patient with type 2 diabetes for 5 years, with a BMI of 33.6 kg/m², after an acute coronary event, with an ejection fraction of 45%, previously treated with a combination preparation: metformin + sitagliptin at a daily dose of 1000+50 mg/day, HbA1c level is 6.8%. Indicate the correct course of action:	A. Glucose levels are normal, continuation of the current treatment is appropriate.	C	C	C	5	5
		B. The glycated hemoglobin level is suboptimal, the dose of metformin should be increased to 3000 mg/day.					
		C. A change in therapy is necessary in the form of; still metformin and the implementation of drugs with proven beneficial cardiovascular effects: empagliflozin and due to obesity semaglutide.					
		D. Due to the slightly elevated glycated hemoglobin level, it is necessary to modify the therapy and implement triple therapy; increase the dose of metformin to 3000 mg/day, sitagliptin 100 mg/day, and gliclazide 60 mg/day.					
		E. Sitagliptin should be discontinued and a sulfonylurea derivative should be introduced.					
51	Which of the following drugs should be used in a normotensive patient with chronic kidney disease due to glomerulonephritis, eGFR 31 ml/min/1.73 m2, with nephrotic syndrome, after a myocardial infarction 4 years ago, with HbA1c 6.3%, treated with metformin 2×850 mg, in addition to ramipril 5.0 mg, indapamide 1.5 mg, atorvastatin 40 mg, nebivolol 5 mg, alfacalcidol 0.25 µg:	A. Continue metformin, introduce ramipril 2.5 mg.	C	C	C	5	5
		B. Continue metformin, introduce fenofibrate 160 mg and dapagliflozin 10 mg.					
		C. Reduce the dose of metformin, introduce dapagliflozin 10 mg and finerenone 10 mg.					
		D. Discontinue metformin.					
		E. The therapy does not require changes.					
52	A patient after an acute coronary event, with newly diagnosed diabetes, is being discharged from the hospital. In this situation, it is necessary to implement:	A. Acetylsalicylic acid.	D	D	D	5	5
		B. Sitagliptin.					
		C. Gliclazide.					
		D. Empagliflozin.					
		E. Pioglitazone.					
53	A patient with newly diagnosed type 2 diabetes should be advised to implement nutritional therapy. Indicate the false statement:	A. In most people with diabetes, as in the general population, the proportion of energy from protein in the diet should be 15-20% (about 1-1.5 g/kg body weight/day).	X	X	C	X	X
		B. A calorie intake from carbohydrates lower than 45% (25-44%) may be temporarily recommended for people with low physical activity, whose possibilities of increasing it are limited.					
		C. In people with type 2 diabetes with excessive body weight, a reduced-calorie diet containing 50% protein should be implemented, which provides greater satiety and facilitates the reduction and maintenance of a normal body weight.					
		D. People with chronic kidney disease should maintain a protein intake of about 0.8-1 g/kg body weight/day.					
		E. Dietary recommendations should not be implemented.					
54	A 49-year-old patient with type 1 diabetes for 16 years, complicated by diabetic eye disease, polyneuropathy, presented to a diabetologist due to pain in the medial malleolus of the right foot. An X-ray of the foot performed during a stay in the ER showed no changes in the bone structures. The foot is without swelling, with moderately increased warmth in the painful area. Indicate the correct course of action:	A. Compresses and NSAIDs.	C	C	C	5	5
		B. Antibiotic therapy for 21 days.					
		C. Immobilization of the foot, e.g., orthosis, cast, and an MRI order.					
		D. Referral for rehabilitation.					
		E. Consultation with a vascular surgeon.					
55	A 25-year-old patient with type 1 diabetes was admitted to the ER at night in a serious general condition. Intensive insulin therapy used: lispro and degludec insulin, the last dose of this insulin was administered in the evening. In the history taken from the parents: nausea, vomiting, deterioration of general condition. The patient did not eat, did not use lispro insulin. Body weight 63 kg, height 158 cm, in laboratory tests: arterial blood pH 6.85, glucose concentration 511 mg/dl, K 5.1 mmol/l. Which of the following treatment schemes is correct (drugs administered intravenously)?:	A. Intravenous infusion of insulin 6 U/h, 80 mmol NaHCO3, 10 mmol KCl.	B	E	B	5	5
		B. Intravenous infusion of insulin 6 U/h, 40 mmol NaHCO3, 10 mmol KCl.					
		C. 6 U of insulin in a bolus, 100 mmol NaHCO3, 20 mmol KCl.					
		D. 6 U of insulin in a bolus, 20 mmol NaHCO3, 30 mmol KCl.					
		E. 6 U of insulin in a bolus, 80 mmol NaHCO3, 10 mmol KCl.					
56	A 63-year-old patient with type 2 diabetes presented to a diabetologist with a painful, small ulcer on the sole of the foot in the heel area. Due to the pain, he is unable to bear weight on the heel and does not allow the wound to be debrided without anesthesia. Indicate the correct course of action:	A. Total contact cast.	D	C	E	4	5
		B. Anesthesia, wound debridement, and silver dressings.					
		C. Assessment of probing to bone, silver dressing.					
		D. Urgent vascular consultation.					
		E. Anesthesia, wound debridement, and dressings with TLC-NOSF technology.					
57	As a supplement to the treatment of neuropathic pain in a patient using a full dose of pregabalin, the following is not used:	A. Gabapentin.	A	A	A	5	5
		B. Paracetamol.					
		C. Acupuncture.					
		D. Amitriptyline.					
		E. Alpha-lipoic acid.					
58	The faster action of FIASP insulin was achieved by adding nicotinamide. How was the stability of the solution improved?:	A. Proline was added to position 30 of the B chain.	D	D	D	5	5
		B. Lysine was inserted at position 7 of the A chain.					
		C. Glutamine was inserted at position 21 of the A chain.					
		D. Arginine was added to the solution.					
		E. Calcium ions were added to the solution.					
59	In addition to zinc ions, an important role in the formation of insulin hexamers is played by the presence of:	A. Phenylalanine.	E	E	E	5	5
		B. Cresol.					
		C. Alanine.					
		D. Pyridoxine.					
		E. Phenol.					
60	A 66-year-old patient, a retired accountant, with type 2 diabetes, recently treated with gliclazide (60 mg) and metformin (2 g), presented to the diabetic clinic due to persistent hyperglycemia. At the time of diagnosis, body weight was 125 kg, with a reduction of over 25 kg since then. In laboratory tests, HbA1c 9.8%, fasting glucose 253 mg/dl. In the glucometer, glucose levels of 300-400 mg/dl. Based on data from the P1 platform, prescription fulfillment was positively assessed. How should the treatment be modified?:	A. Introduce an SGLT2 preparation.	E	E	E	5	5
		B. Introduce semaglutide.					
		C. Introduce pioglitazone.					
		D. Introduce a gliptin.					
		E. Start insulin therapy.					
61	The receptors responsible for intestinal glucose absorption are:	A. GLUT 1.	B	B	B	5	5
		B. GLUT 2.					
		C. GLUT 3.					
		D. GLUT 4.					
		E. GLUT 5.					
62	A 69-year-old patient with long-standing type 1 diabetes, treated for painful polyneuropathy, complains of persistent sneezing, which he associates with the start of therapy. After which substance can such an adverse effect be expected:	A. Capsaicin.	A	C	A	5	5
		B. Gabapentin.					
		C. Duloxetine.					
		D. Carbamazepine.					
		E. Amitriptyline.					
63	To calculate the insulin action index, it is necessary to perform:	A. An oral glucose tolerance test (OGTT).	C	D	D	5	5
		B. A measurement of fasting glucose and insulin.					
		C. An intravenous glucose tolerance test (IVGTT).					
		D. A euglycemic metabolic clamp.					
		E. Himsworth's test.					
64	A 57-year-old man with type 2 diabetes for 10 years presented to the diabetic clinic for a control examination. In his history: hypertension, dyslipidemia (currently TG 220 mg/dl, LDL cholesterol 65 mg/dl), overweight (BMI 28 kg/m2), HbA1c 7.0%, ambulatory blood pressure <130/80 mmHg. Previous treatment: metformin 3×1000 mg, empagliflozin, GLP-1 agonist at the maximum dose, ACEI, β-blocker, atorvastatin 80 mg. The patient should be offered: (1) adding a sulfonylurea derivative to the treatment; (2) maintaining the currently used antihyperglycemic and hypotensive drugs; (3) reducing the dose of statin; (4) adding a fibrate to the current treatment; (5) adding acetylsalicylic acid to the current treatment. The correct answer is:	A. All of the above.	C	C	B	5	5
		B. 4, 5.					
		C. 2, 4.					
		D. 2, 3, 4.					
		E. 2, 3.					
65	Which of the following substances can interfere with the glucose reading when using the Dexcom G6 CGM system? (1) alcohol; (2) ascorbic acid; (3) mannitol; (4) tetracycline; (5) hydroxyurea. The correct answer is:	A. 1, 2, 4, 5.	C	D	C	4	5
		B. 1, 3, 5.					
		C. Only 5.					
		D. 2, 4.					
		E. 1, 2.					
66	In a patient with type 2 diabetes (currently HbA1c 6.2%), ischemic heart failure with preserved ejection fraction (HFpEF), previously treated with metformin 3×1000 mg, sitagliptin 100 mg 1×1 tab., empagliflozin 10 mg 1×1 tab., chronic kidney disease with a glomerular filtration rate (eGFR) of 18 ml/min/1.73 m2 was diagnosed. The patient's antihyperglycemic treatment should be modified as follows:	A. Discontinue metformin, reduce sitagliptin to 1×25 mg, continue empagliflozin.	A	B	B	5	5
		B. discontinue metformin and empagliflozin, reduce sitagliptin to 1×25 mg.					
		C. Discontinue metformin, reduce sitagliptin to 1×50 mg, continue empagliflozin.					
		D. Use a GLP-1 agonist and discontinue sitagliptin, metformin, and empagliflozin.					
		E. Reduce the dose of metformin to 2×500 mg and sitagliptin to 1×50 mg, leave empagliflozin.					
67	A 76-year-old woman, after a myocardial infarction, with hypertension, chronic kidney disease stage G4 (eGFR 20 ml/min/1.73 m2), stage I obesity, with type 2 diabetes for 20 years. Previous treatment: atorvastatin 20 mg, perindopril 10 mg, hydrochlorothiazide 12.5 mg, bisoprolol 2.5 mg, metformin extended-release 500 mg, dapagliflozin 10 mg, semaglutide 0.5 mg. In laboratory tests: HbA1c 7.7%, LDL 2.4 mmol/l, non-HDL 3.2 mmol/l, triglycerides 1.6 mmol/l. Blood pressure at home <140/80 mmHg. In this patient, it is necessary to: (1) discontinue metformin; (2) increase the dose of atorvastatin; (3) increase the dose of semaglutide; (4) switch the thiazide diuretic to a loop diuretic; (5) discontinue dapagliflozin. The correct answer is:	A. 1, 5.	C	C	C	5	5
		B. 1, 4, 5.					
		C. 1, 2, 4.					
		D. 2, 3, 5.					
		E. 1, 3.					
68	Which of the following substances can interfere with the glucose reading when using the Eversense CGM system? (1) alcohol; (2) ascorbic acid; (3) mannitol; (4) tetracycline; (5) hydroxyurea. The correct answer is:	A. 1, 2, 4.	B	C	C	5	5
		B. 3, 4.					
		C. 2, 5.					
		D. 2, 4.					
		E. 3, 5.					
69	A holistic approach to cardiorenal risk includes: (1) the basis is the patient's physical activity and weight reduction; (2) smoking cessation is as important as physical activity; (3) the first-choice drugs in patients with type 2 diabetes are metformin and flozins; (4) in patients whose treatment goals are not achieved, it is recommended to add a GLP-1 agonist with proven cardiovascular benefits to the treatment; (5) the use of a non-steroidal mineralocorticoid receptor antagonist with proven beneficial cardiovascular and nephroprotective effects should be considered in patients with type 2 diabetes with an eGFR ≥25 ml/min/1.73 m2. The correct answer is:	A. All of the above.	A	A	A	5	5
		B. 1, 2, 4, 5.					
		C. 2, 4, 5.					
		D. 1, 4.					
		E. 1, 5.					
70	A 30-year-old patient with type 1 diabetes, after completing treatment for Graves' disease 5 years ago, is planning a pregnancy. What laboratory tests should be performed on the patient? (1) HbA1c; (2) TSH; (3) FT3; (4) FT4; (5) TRAb. The correct answer is:	A. 1, 3, 4, 5.	B	B	B	5	5
		B. 1, 2, 4, 5.					
		C. 1, 2.					
		D. 2, 5.					
		E. 2, 3, 4.					
71	In a patient with type 2 diabetes and heart failure (HF), it is necessary to: (1) use antihyperglycemic drugs with proven cardioprotective effects; (2) systematically measure the concentration of natriuretic peptides, and if their concentration is normal, refrain from diagnosing HF; (3) in heart failure with reduced ejection fraction, consider the use of a neprilysin inhibitor as first-line treatment; (4) in a situation of no heart rhythm control in atrial fibrillation (>75 beats/minute), use ivabradine; (5) use resynchronization therapy in patients with diabetes and atrial fibrillation. The correct answer is:	A. 2, 3.	C	C	C	5	5
		B. 1, 5.					
		C. 1, 3.					
		D. 3, 5.					
		E. 1, 4.					
72	The principles of physical exercise for people treated with a hybrid closed-loop system include: (1) consuming carbohydrates up to 5-10 minutes before exercise, but entering only 50% of the consumed carbohydrates into the system; (2) consuming carbohydrates up to 5-10 minutes before exercise, but without entering them into the system; (3) consuming carbohydrates up to 30 minutes before exercise, but entering only 50% of the consumed carbohydrates into the system; (4) consuming carbohydrates up to 30 minutes before exercise, but without entering them into the system; (5) meals consumed up to 2 hours before exercise require a 25-75% reduction in the insulin dose. The correct answer is:	A. 1, 5.	E	E	E	5	5
		B. 1, 4.					
		C. 1, 3.					
		D. 2, 4, 5.					
		E. 2, 5.					
73	Which of the following drugs and chronic diseases can be a secondary cause of hyperlipidemia? (1) systemic lupus erythematosus; (2) psoriasis; (3) duloxetine; (4) thiazide diuretics; (5) sulfonylurea derivatives. The correct answer is:	A. All of the above.	D	D	D	5	5
		B. 1, 3, 4.					
		C. 2, 3, 5.					
		D. 1, 2, 4.					
		E. 2, 4.					
74	Indicate the false statements regarding the prevention, diagnosis, and treatment of diabetic foot syndrome: (1) if a monofilament and tuning fork are not available, it is possible to examine the patient's feet by lightly touching the patient's toes with the examiner's index finger for 2-3 seconds; (2) antibiotic therapy should be used for 3 weeks in the case of osteomyelitis if the bones affected by the inflammatory process have not been removed or amputation has not been performed; (3) SINBAD is a classification in which ischemia is assessed based on a palpable pulse in at least one artery and clinical features of ischemia; (4) the PEDIS classification is recommended for the assessment of the diabetic foot; (5) the diagnosis of Charcot neuro-osteoarthropathy should begin with an X-ray of the foot in a lying position. The correct answer is:	A. 1, 2.	D	B	A	5	5
		B. 1, 3.					
		C. 1,4.					
		D. 2, 5.					
		E. 3, 4, 5.					
75	Indicate the true statements regarding exercise-induced hypoglycemia in patients with type 1 diabetes: (1) if hyperglycemia >250 mg/dl is not accompanied by ketonuria and/or ketonemia, and/or the cause of hyperglycemia is known, then light to moderate aerobic exercise can be undertaken, but mixed (aerobic-anaerobic) exercise cannot be undertaken; (2) anaerobic exercise can cause hyperglycemia, the correction of which with fast-acting insulin should be cautious due to the risk of hypoglycemia appearing several hours after the end of physical exercise; (3) in the case of severe hypoglycemia in a person with type 1 diabetes, the effect of glucagon after intense physical exercise is not effective and this drug should not be administered; (4) in the case of a hypoglycemia alert ≤70 mg/dl, simple carbohydrates should be consumed, preferably in liquid form, and physical exercise can be continued after the symptoms of hypoglycemia have subsided; (5) after a sprint, an increase in glucose levels should be expected, and the risk of hypoglycemia is small. The correct answer is:	A. 2, 4, 5.	X	X	X	X	X
		B. 1, 2.					
		C. 2, 4.					
		D. 1, 2, 3, 4.					
		E. 1, 3, 4, 5.					
76	A 75-year-old patient with long-standing type 1 diabetes presented for a control visit to the diabetic clinic with symptoms of intermittent claudication and a forefoot wound involving all soft tissues with signs of inflammation not exceeding 1.5 cm from the ulcer border. The patient is afebrile. In the TcpO2 test, the value is 35 mmHg. What is the stage of the patient's diabetic foot syndrome in the PEDIS classification?:	A. 1.	B	D	B	5	5
		B. 2.					
		C. 3.					
		D. 4.					
		E. Ulcers are not assessed in the PEDIS classification.					
77	A 75-year-old patient with long-standing type 1 diabetes, after a heart transplant 10 years ago, presented for a control visit to the diabetic clinic with symptoms of intermittent claudication and a forefoot wound involving all soft tissues with signs of inflammation not exceeding 1.5 cm from the ulcer border. The patient is afebrile. In this patient, it is necessary to apply:	A. Antibiotic therapy only in the case of a confirmed infection, for the period of wound healing.	E	E	E	5	5
		B. antibiotic therapy only in the case of a confirmed infection, taking into account the presence of Gram-negative bacteria, anaerobes.					
		C. Antibiotic therapy for 8 weeks in the case of osteomyelitis, if the bones affected by the inflammatory process have not been removed.					
		D. Antibiotic therapy for 6 weeks after amputation performed due to osteomyelitis.					
		E. Antibiotic therapy for at least 2 weeks, but longer use should be considered due to immunosuppression.					
78	In a patient with type 2 diabetes, with a moderate cardiovascular risk, well metabolically controlled, who has been using atorvastatin at a dose of 20 mg/day for 6 months, and lipid concentrations are: LDL cholesterol 110 mg/dl, HDL-cholesterol 35 mg/dl, triglycerides 196 mg/dl, it is necessary to apply:	A. Atorvastatin 20 mg with a fibrate.	C	C	C	5	5
		B. Atorvastatin 20 mg with ezetimibe 10 mg.					
		C. Atorvastatin at a dose of 40-80 mg/day.					
		D. Rosuvastatin 20 mg with fenofibrate.					
		E. Rosuvastatin 40 mg with ezetimibe 10 mg.					
79	Which of the following combinations of oral drugs is not indicated in a 54-year-old ventilation systems installer?:	A. Metformin + sitagliptin.	B	B	B	5	5
		B. Metformin + gliclazide.					
		C. Metformin + sitagliptin + dapagliflozin.					
		D. Linagliptin + dapagliflozin.					
		E. Vildagliptin + empagliflozin.					
80	In routine fasting laboratory tests of a 34-year-old obese man, a musician by profession (brass instruments), who uses a wheelchair due to post-traumatic lower limb paresis, treated for rheumatoid arthritis with methotrexate and methylprednisolone, and who does not report symptoms of diabetes, a glucose level of 144 mg/dl was found. Indicate the further course of action:	A. Recommendation to increase physical activity.	C	C	C	5	5
		B. Perform a prolonged 3-hour oral glucose tolerance test.					
		C. Measure HbA1c.					
		D. None, because such a glucose value and the absence of diabetes symptoms are typical during steroid therapy.					
		E. None, because such a glucose value in a sick person with a poor overall prognosis does not require intervention.					
81	Which of the following actions does not affect the risk of developing diabetes in a 55-year-old obese dairy worker who was found to have a fasting glucose level of 105 mg/dl on one occasion?:	A. Swimming for 1 hour twice a week.	C	C	C	5	5
		B. 3-hour cycles of resistance training per week.					
		C. Taking a berberine preparation.					
		D. Taking metformin at a dose of 2×850 mg/day.					
		E. Taking semaglutide at a dose of 1 mg/week.					
82	Which of the following adverse effects does not apply to GLP-1 analogs?:	A. Sinus node inhibition.	A	E	A	5	5
		B. Vomiting.					
		C. Constipation.					
		D. Skin changes.					
		E. Sarcopenia.					
83	A 49-year-old owner of a cat food production plant, with type 1 diabetes since the age of 12, using a continuous glucose monitoring system, HbA1c from a month ago 8.9%, eGFR 48 ml/min/1.73 m2, proteinuria, ejection fraction 55%, treated for hypertension with ramipril and amlodipine, after urosepsis 4 years ago, presented to a diabetologist with a request to prescribe a flozin. Should the diabetologist do this?:	A. Yes, because that is the patient's wish.	B	B	E	5	5
		B. Yes, because the patient has chronic kidney disease.					
		C. No, because he uses a continuous glucose monitoring system.					
		D. No, because he had urosepsis.					
		E. No, because the patient has type 1 diabetes.					
84	In whom is there no need to measure the titer of anti-glutamic acid decarboxylase antibodies?:	A. A 22-year-old pregnant mechatronics student, in whom diabetes was diagnosed during screening tests performed during the first visit to a gynecologist in the 7th week of pregnancy.	B	D	C	5	5
		B. A 29-year-old IT specialist with type 1 diabetes since the age of 5, with long-standing autoimmune thyroiditis, hospitalized for ketoacidosis for the first time in his life.					
		C. A 33-year-old carpenter, after traumatic amputation of the left forearm, whose only older sister by 4 years, an endoscopy nurse, has had type 1 diabetes since the age of 23, and who was found to have a fasting glucose level of 103 mg/dl in control tests.					
		D. A 36-year-old driving instructor for categories B and C, BMI 32.2 kg/m2, numerous tattoos on the skin of the trunk and limbs, previously untreated for chronic diseases, hospitalized for significant hyperglycemia and ketoacidosis in the course of acute pancreatitis.					
		E. A 42-year-old captain of the Polish Army, commander of a long-range reconnaissance company, BMI 29.3 kg/m2, with newly diagnosed diabetes without clinical symptoms of hyperglycemia.					
85	A 67-year-old retired violist treated for type 2 diabetes for 12 years, for over 4 years treated with a combination of metformin 3×850 mg/day, empagliflozin 1× 10 mg/day, and semaglutide 1 mg/week on reimbursement, BMI 34.8 kg/m2, HbA1c 6.4%, presented to a diabetologist with a request to switch semaglutide to tirzepatide because she wanted to reduce her body weight more - she has not been losing weight for over a year, and previously she had lost 24 kg in 2 years. Should the doctor use tirzepatide in her case, and if so, how?:	A. Yes, starting with a dose of 2.5 mg/week or 5 mg/week and discontinuing semaglutide.	A	A	A	5	5
		B. Yes, by adding tirzepatide at a dose of 2.5 mg to the current treatment.					
		C. No, because the HbA1c value is within the target range.					
		D. No, because after such a weight reduction, the use of tirzepatide will not bring significant effects.					
		E. No, because the patient will experience a rapid increase in treatment costs and will not continue the incretin therapy.					
86	In a patient with type 2 diabetes treated with metformin, sitagliptin, glimepiride, empagliflozin, and dulaglutide, a rapid progression of chronic kidney disease has recently been noted. Which of the drugs will be able to be used the longest as the glomerular filtration deteriorates, i.e., until the implementation of renal replacement therapy?:	A. Metformin.	E	E	D	5	5
		B. Glimepiride.					
		C. Empagliflozin.					
		D. Dulaglutide.					
		E. Answers C and D are correct.					
87	In which clinical situation is there no indication for the use of a continuous glucose monitoring system? (1) impaired fasting glucose (IFG); (2) newly diagnosed type 1 diabetes in remission; (3) newly diagnosed type 2 diabetes; (4) type 2 diabetes associated with obesity; (5) type 2 diabetes requiring the use of oral drugs; (6) type 2 diabetes requiring the use of basal insulin; (7) diabetes in a person after pancreatic resection; (8) type 2 diabetes in a person over 80 years of age; (9) diabetes complicated by end-stage renal disease - during dialysis therapy; (10) diabetes in the course of chronic pancreatitis. The correct answer is:	A. Only 1.	A	A	B	5	5
		B. 1, 2, 3, 4.					
		C. 2, 5, 8, 9.					
		D. 6, 7, 10.					
		E. 1, 5.					
88	Arrange the insulin preparations in order from the longest-acting to the shortest-acting preparation:	A. Degludec, glargine U300, glargine U100, detemir.	A	A	A	5	5
		B. Degludec, glargine U100, glargine U300, detemir.					
		C. Degludec, glargine U300, detemir, glargine U100.					
		D. Glargine U300, degludec, detemir, glargine U100.					
		E. Glargine U300, degludec, glargine U100, detemir.					
89	From what value (in mmol/l) of beta-hydroxybutyrate concentration in the blood is diabetic ketoacidosis diagnosed?:	A. >2.	B	A	B	4	5
		B. >3.					
		C. >4.					
		D. >5.					
		E. >6.					
90	Which of the following drugs is not used in the treatment of diabetic polyneuropathy?:	A. Sildenafil.	A	A	A	5	5
		B. Venlafaxine.					
		C. Amitriptyline.					
		D. Buprenorphine.					
		E. Tramadol.					
91	According to the PTD recommendations, how often should a person with diabetes have a dental examination:	A. At the diagnosis of diabetes, then as needed, but no less often than every 5 years.	C	C	C	5	5
		B. In well-controlled diabetes every 5 years, in metabolically uncontrolled every 6 months.					
		C. Once a year.					
		D. In case of complaints or the need to fit a prosthesis.					
		E. Within 6-8 weeks after a serious cardiovascular event.					
92	A 59-year-old locksmith-welder, a manufacturer of chain barriers, with diabetes for almost 20 years, for several years has only been using intensive insulin therapy (lispro 3×20-24 U + glargine U300 40 U before bed), BMI 36.1 kg/m2, HbA1c 9.8%, eGFR 58 ml/min/1.73 m2. The addition of which drug to insulin therapy is not justified at this stage of diabetes:	A. Metformin.	D	D	D	5	5
		B. Tirzepatide.					
		C. Dulaglutide.					
		D. Sitagliptin.					
		E. Dapagliflozin.					
93	In diabetic ketoacidosis, the following is not routinely administered intravenously:	A. Glucose.	C	C	C	5	5
		B. Insulin.					
		C. Bicarbonates.					
		D. Potassium.					
		E. Sodium chloride.					
94	A contraindication to participating in training is persistent hyperglycemia for 2 hours above:	A. 180 mg/dl (10 mmol/l).	E	D	D	5	5
		B. 200 mg/dl (11.1 mmol/l).					
		C. 220 mg/dl (12.2 mmol/l).					
		D. 250 mg/dl (13.9 mmol/l).					
		E. 300 mg/dl (16.7 mmol/l).					
95	A characteristic function of an insulin pump system functionally integrated with a continuous glucose monitoring system is:	A. Automatic basal.	B	B	B	5	5
		B. Automatic insulin suspension in the prediction of hypoglycemia based on the values indicated by the CGM system.					
		C. Automatic administration of correction boluses.					
		D. Automatic switching to manual mode in case of CGM system and/or application failure.					
		E. Setting a temporary mode/target for the prevention of hypoglycemia in situations that increase its risk.					
96	A 57-year-old patient with well-controlled type 2 diabetes is being prepared for laparoscopic cholecystectomy. Should and when should the administration of a flozin be stopped in his case?:	A. 10 days before the procedure.	C	B	C	5	5
		B. A week before the procedure.					
		C. 2 days before the procedure.					
		D. In the morning on the day of the procedure.					
		E. There is no need to stop the administration of the drug.					
97	In the diet of a pregnant woman with diabetes, carbohydrates with a low glycemic index are preferred in an amount of approximately:	A. 100 g/day.	E	C	D	5	5
		B. 120 g/day.					
		C. 140 g/day.					
		D. 160 g/day.					
		E. 180 g/day.					
98	Treatment of gestational hypertension should be started at blood pressure values exceeding:	A. 120/80 mmHg.	E	E	E	5	5
		B. 130/80 mmHg.					
		C. 130/85 mmHg.					
		D. 140/80 mmHg.					
		E. 140/90 mmHg.					
99	A classic clinical symptom of Charcot foot is not:	A. Unilateral foot swelling.	C	C	C	5	5
		B. Weakened vibration sensation in the foot.					
		C. Ulceration on the foot.					
		D. Redness of the foot.					
		E. Increased warmth of the foot.					
100	The second-line drug in the treatment of painful diabetic neuropathy is:	A. Amitriptyline.	D	E	D	5	5
		B. Duloxetine.					
		C. Pregabalin.					
		D. Tramadol.					
		E. Venlafaxine.					
101	In the case of vitreoretinal traction running vertically towards the macula, the treatment of choice is:	A. Bevacizumab injection.	D	D	D	5	5
		B. Micropulse laser therapy.					
		C. Grid laser therapy.					
		D. Vitrectomy.					
		E. Use of sulodexide.					
102	A 66-year-old man with type 2 diabetes and chronic kidney disease stage G4A2 (eGFR 28 ml/min/1.73 m2 and ACR 254 mg/g creatinine) has not previously been treated with a sodium-glucose cotransporter 2 inhibitor (SGLT2i). After starting the drug, monitoring of creatinine concentration:	A. Is not required.	A	D	D	5	5
		B. Should be performed after a month.					
		C. Should be performed every 3 months.					
		D. Should be performed in the sequence - after a month, after 3 months, and every six months.					
		E. In this patient, an SGLT2i should not be added to the therapy.					
103	Indicate the optimal therapy model for heart failure with LVEF ≤40% (HFrEF), in a 70-year-old woman with an HbA1c of 6.4%:	A. ACEI, ARNI, β-blocker, MRA.	D	D	D	5	5
		B. ACEI, ARB, β-blocker, SGLT2i.					
		C. ACEI, ARNI, MRA, SGLT2i.					
		D. ARNI, β-blocker, MRA, SGLT2i.					
		E. ARB, ARNI, MRA, SGLT2i.					
104	A risk factor for chronic coronary syndrome in a person with diabetes is not the presence of:	A. Albuminuria.	D	B	B	5	5
		B. Enteropathy.					
		C. Gastroparesis.					
		D. Diabetic polyneuropathy.					
		E. Sweating disorders.					
105	The corrected natremia of a patient admitted with hyperglycemia of 897 mg/dl (50 mmol/l), whose current sodium concentration determined in the laboratory is 128 mmol/l, is:	A. 120 mmol/l.	D	D	D	4	5
		B. 128 mmol/l.					
		C. 136 mmol/l.					
		D. 144 mmol/l.					
		E. 152 mmol/l.					
106	In a patient using bempedoic acid, one should expect the possibility of:	A. Muscle pain.	B	B	B	5	5
		B. Symptoms of gout.					
		C. Fatigue.					
		D. Significantly elevated liver transaminase activity.					
		E. Significantly elevated creatine kinase concentration.					
107	In a patient with type 2 diabetes and hypertension who has overt proteinuria (A3), blood pressure should be lowered to:	A. 110/65 mmHg.	C	C	C	5	5
		B. 120/70 mmHg.					
		C. 130/80 mmHg.					
		D. 140/80 mmHg.					
		E. 140/90 mmHg.					
108	A 19-year-old man with type 1 diabetes is under the care of a dermatologist for acne. The doctor suggested a long-term course of tetracycline. Which continuous glucose monitoring system should not be recommended for this patient during therapy?:	A. Dexcom G6.	D	D	D	5	5
		B. FreeStyle Libre2.					
		C. Medtronic Guardian4.					
		D. Senseonics Eversense.					
		E. The use of tetracycline should not interfere with the glucose reading of any of the listed CGM systems.					
109	A 50-year-old patient referred from a primary care clinic, with type 2 diabetes for 10 years, currently treated with a combination drug sitagliptin + metformin 50/1000 mg 2× daily, HbA1c 7.2%, BMI 30.7 kg/m2, without heart failure, creatinine 1.28 mg/dl (113.15 μmol/l), eGFR 68 ml/min/1.73 m2, trace protein in general urinalysis, ACR 37.3 mg/g, ultrasound shows metabolic-associated steatotic liver disease (MASLD). What treatment should be proposed?:	A. Add an SGLT-2 inhibitor.	B	A	B	5	5
		B. Add a GLP-1 receptor agonist in place of sitagliptin.					
		C. Add a sulfonylurea derivative.					
		D. Add pioglitazone.					
		E. Maintain the current treatment - the patient does not require therapy intensification.					
110	Which of the hypoglycemic drugs requires dose modification when eGFR drops to 25 ml/min/1.73 m2 (stage G4)?:	A. Empagliflozin.	B	B	B	5	5
		B. Sitagliptin.					
		C. Pioglitazone.					
		D. Semaglutide.					
		E. None of the above requires a dose reduction in stage G4 chronic kidney disease.					
111	In a patient with type 2 diabetes and heart failure with reduced ejection fraction (HFrEF), the use of the following is contraindicated:	A. Gliclazide.	C	C	C	5	5
		B. Empagliflozin.					
		C. Pioglitazone.					
		D. Semaglutide.					
		E. Metformin.					
112	A 40-year-old woman with a history of gestational diabetes 8 years ago, with newly diagnosed type 2 diabetes - fasting glucose twice >126 mg/dl (>7.0 mmol/l), with stage II obesity (BMI 36.4 kg/m2), hypertension, and dyslipidemia was referred to a diabetologist by a primary care physician. In the attached tests: HbA1c 6.2%, creatinine 0.82 mg/dl (72.5 μmol/l), eGFR 96 ml/min/1.73 m2, ACR 12.2 mg/g, calculated SCORE2-Diabetes index 2.9%. According to clinical recommendations, she can be treated with: (1) metformin; (2) SGLT-2 inhibitor; (3) semaglutide 1.0 mg; (4) liraglutide 1.2-3.0 mg; (5) tirzepatide 5-15 mg. The correct answer is:	A. All of the above.	A	B	A	5	5
		B. 1, 3, 4, 5.					
		C. 1, 2, 4, 5.					
		D. 1, 2, 3, 5.					
		E. 2, 3, 4.					
113	A 27-year-old patient in her first pregnancy had her fasting glucose measured in the first trimester and presented with a result of 99 mg/dl (5.5 mmol/l). In this patient, it is necessary to:	A. Diagnose gestational diabetes and immediately implement appropriate treatment.	C	D	A	4	5
		B. Perform a repeat fasting glucose measurement.					
		C. Perform an oral glucose tolerance test urgently.					
		D. Perform an oral glucose tolerance test between the 24th and 28th week of pregnancy.					
		E. Measure the percentage of glycated hemoglobin to confirm the diagnosis of gestational diabetes.					
114	Cardiovascular benefits in prospective randomized clinical trials have been demonstrated for the following, except:	A. Dapagliflozin.	D	D	D	5	5
		B. Metformin.					
		C. Semaglutide.					
		D. Insulin.					
		E. Empagliflozin.					
115	A 61-year-old patient with long-standing type 1 diabetes after a recent myocardial infarction presented for a follow-up visit at the diabetic clinic. The patient was previously treated with a rapid-acting analog for meals and NPH insulin before bed, and used a glucometer for self-monitoring. During his hospital stay, heart failure with reduced ejection fraction (EF 35%) was diagnosed and an SGLT-2 inhibitor, ACE inhibitor, metoprolol XR, eplerenone, and torsemide were recommended. During hospitalization, episodes of hypoglycemia at night were noticed in the patient. In the provided tests, HbA1c 6.3%, glucosuria, overt proteinuria, creatinine 1.38 mg/dl (122.0 μmol/l), eGFR 58 ml/min/1.73 m2, potassium concentration 5.9 mmol/l. In this patient, it is necessary to: (1) immediately discontinue the SGLT-2 inhibitor (risk of ketoacidosis); (2) discontinue eplerenone; (3) recommend the use of CGM; (4) switch NPH insulin to a long- or ultra-long-acting analog (degludec or glargine); (5) recommend a nephrology consultation. The correct answer is:	A. 1, 2, 4, 5.	B	B	B	5	5
		B. 2, 3, 4, 5.					
		C. 1, 3, 4, 5.					
		D. 1, 2, 3, 5.					
		E. 1, 2, 3, 4.					
116	Diabetic neuropathy is a common complication in both types of diabetes. In the treatment of painful diabetic neuropathy, the following is not used:	A. Pregabalin.	E	E	E	5	5
		B. Tramadol.					
		C. Duloxetine.					
		D. Venlafaxine.					
		E. All of the above are used in the treatment of painful neuropathy.					
117	A 62-year-old woman treated with intensive insulin therapy combined with metformin (2×1000 mg) for uncontrolled type 2 diabetes diagnosed 12 years ago presented to the diabetic clinic. She is also treated for hypertension (currently using a sartan, calcium antagonist, β-blocker, and thiazide-like diuretic) and dyslipidemia. Body weight in the overweight range. The patient was concerned about the results of recent tests, in which massive proteinuria appeared in the urine test, and in blood tests: HbA1c 8.3%, creatinine 1.43 mg/dl (126.4 μmol/l), eGFR 41 ml/min/1.73 m2, potassium 4.7 mmol/l. In this patient, it is necessary to:	A. Add an SGLT-2 inhibitor to the treatment.	E	D	E	5	5
		B. Add a mineralocorticoid receptor antagonist (finerenone).					
		C. Reduce the dose of metformin by half.					
		D. Answers A and B are correct.					
		E. Answers A, B, and C are correct.					
118	One of the manifestations of endocrine diseases can be diabetes. Endocrine system diseases that can lead to hyperglycemia do not include:	A. Glucagonoma.	E	E	E	5	5
		B. Pheochromocytoma of the adrenal glands.					
		C. Acromegaly.					
		D. Cushing's disease.					
		E. Addison's disease.					
119	A 37-year-old patient with long-standing type 1 diabetes presented for a follow-up visit. Recently, she has noticed periodically worsening gastrointestinal complaints (abdominal pain, bloating), especially after starchy foods. Laboratory tests show mild iron deficiency anemia (despite not very heavy menstruation). A fecal occult blood test was negative (repeated twice). In the differential diagnosis, the following should be considered:	A. Autonomic gastrointestinal neuropathy.	B	B	B	5	5
		B. Celiac disease.					
		C. Addison's disease.					
		D. Crohn's disease.					
		E. All of the above are possible, additional tests are necessary.					
120	Type 2 diabetes is associated with an increased risk of malignant neoplasms. An antidiabetic drug that also has an oncoprotective effect is:	A. Insulin glargine.	C	C	C	5	5
		B. Glimepiride.					
		C. Metformin.					
		D. Sitagliptin.					
		E. None of the above.					
